# Balancing Strength and Flexibility: How the Synthesis, Organization, and Modification of Guard Cell Walls Govern Stomatal Development and Dynamics

**DOI:** 10.3389/fpls.2018.01202

**Published:** 2018-08-20

**Authors:** Yue Rui, Yintong Chen, Baris Kandemir, Hojae Yi, James Z. Wang, Virendra M. Puri, Charles T. Anderson

**Affiliations:** ^1^Department of Biology, The Pennsylvania State University, University Park, PA, United States; ^2^Intercollege Graduate Degree Program in Plant Biology, The Pennsylvania State University, University Park, PA, United States; ^3^Intercollege Graduate Degree Program in Molecular Cellular and Integrative Biosciences, The Pennsylvania State University, University Park, PA, United States; ^4^College of Information Sciences and Technology, The Pennsylvania State University, University Park, PA, United States; ^5^Department of Agricultural and Biological Engineering, The Pennsylvania State University, University Park, PA, United States

**Keywords:** guard cells, plant cell wall, stomatal development, stomatal function, pectin, hemicellulose, cellulose

## Abstract

Guard cells are pairs of epidermal cells that control gas diffusion by regulating the opening and closure of stomatal pores. Guard cells, like other types of plant cells, are surrounded by a three-dimensional, extracellular network of polysaccharide-based wall polymers. In contrast to the walls of diffusely growing cells, guard cell walls have been hypothesized to be uniquely strong and elastic to meet the functional requirements of withstanding high turgor and allowing for reversible stomatal movements. Although the walls of guard cells were long underexplored as compared to extensive studies of stomatal development and guard cell signaling, recent research has provided new genetic, cytological, and physiological data demonstrating that guard cell walls function centrally in stomatal development and dynamics. In this review, we highlight and discuss the latest evidence for how wall polysaccharides are synthesized, deposited, reorganized, modified, and degraded in guard cells, and how these processes influence stomatal form and function. We also raise open questions and provide a perspective on experimental approaches that could be used in the future to shed light on the composition and architecture of guard cell walls.

## Introduction

**Figure 1:**
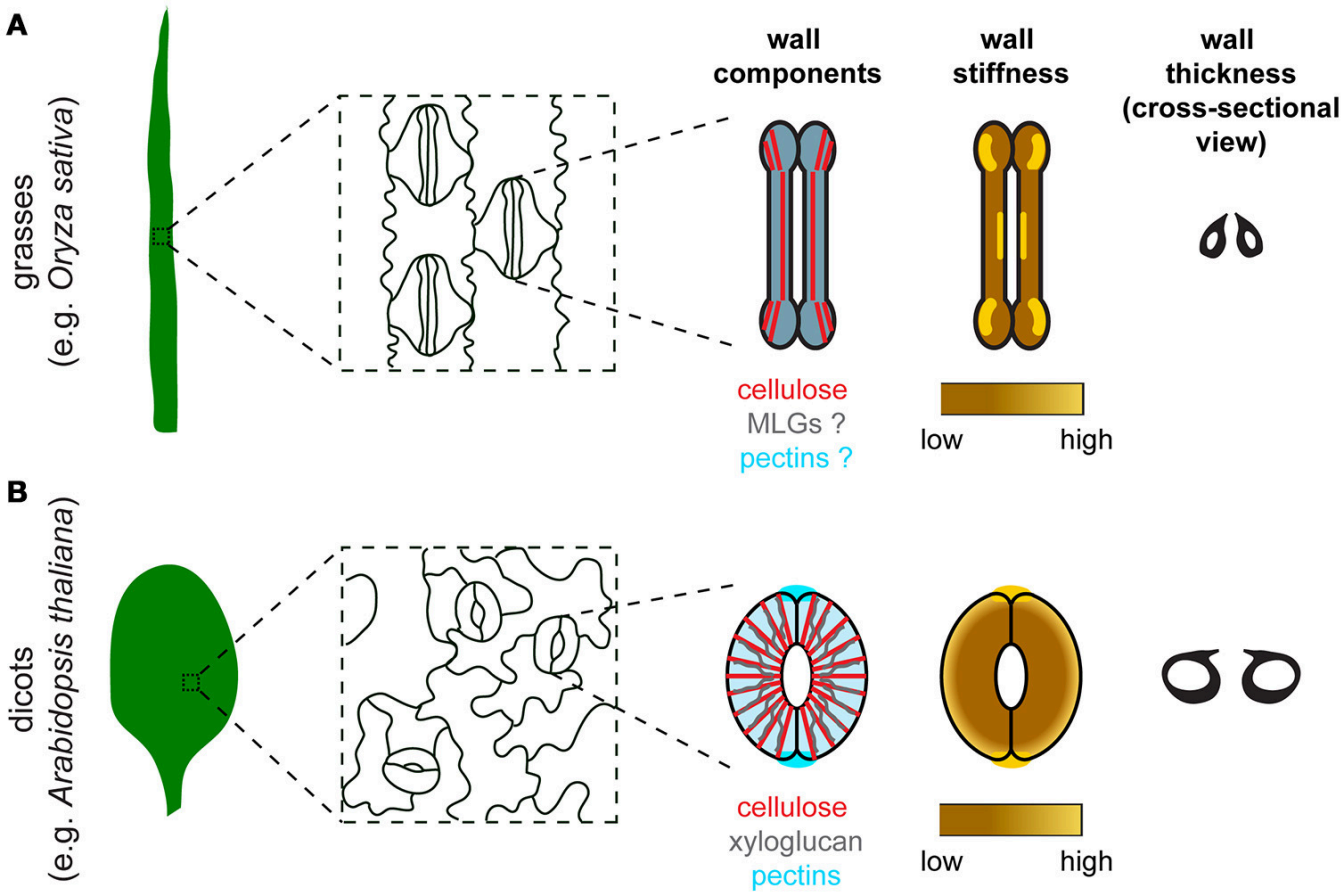
Comparison of stomatal morphology, components, organization, stiffness distribution, and thickness of the guard cell wall between grasses and dicots. **(A)** In grasses such as *Oryza sativa*, guard cells are dumbbell-shaped and stomata are oriented in the same direction in the leaf epidermis. Major components of the guard cell wall in grasses include cellulose (red), mixed-linkage glucans (MLGs, gray), and pectins (blue). Cellulose organization is depicted based on polarized light microscopy data in Shtein et al. (2017). Since the organization of MLGs and pectins in guard cells is unknown, these two components are depicted to have a uniformal distribution with a merged color. Although experimental data to measure guard cell wall stiffness is missing, a speculated stiffness map is provided based on the distribution of crystalline cellulose in guard cells (Shtein et al., 2017). **(B)** In dicots such as *Arabidopsis thaliana*, guard cells are kidney-shaped and stomata are oriented randomly in the leaf epidermis. Major components of the guard cell wall in dicots include cellulose (red), xyloglucan (gray), and pectins (blue). Note that pectic HG is also enriched at the polar ends of guard cell pairs, which coincides with the high stiffness detected in the same regions. High stiffness is concentrated at the polar ends of guard cell pairs based on published AFM data (Carter et al., 2017). Differential wall thickness is illustrated in cross-sectional views of stomata in both grasses **(A)** and dicots **(B)**.

One of the most crucial adaptations for plants to colonize land is the innovation of stomata over 400 million years ago (Edwards et al., 1992; Berry et al., 2010). With an earlier appearance than vascular tissues and roots (Peterson et al., 2010; Chen et al., 2017), stomata are thought to have evolved once (Raven, 2002) and exist in almost all terrestrial plants except liverworts, although some liverwort species have a 16-cell barrel-shaped structure called the air pore complex that might serve a function similar to stomata (Jones and Dolan, 2017). The myriad programs of epidermal growth and development adopted by different species result in a diversity of stomatal ontogeny (Rudall et al., 2013). For example, in some moss species such as *Physcomitrella patens* and *Funaria hygrometrica*, guard mother cells undergo incomplete cytokinesis, which results in a single guard cell encasing a stomatal pore (Sack and Paolillo, 1985; Chater et al., 2016). In vascular plants, stomatal guard cells exist in pairs: grass species typically have two dumbbell-shaped guard cells flanked by specialized subsidiary cells, and their stomata exist in a developmental gradient along the proximodistal leaf axis, which is convergently analogous to progressive stomatal development in hornwort sporophytes (Renzaglia et al., 2017). In contrast, guard cells in most eudicots are kidney-shaped without surrounding subsidiary cells, and the stomata in eudicot leaves vary in age and are oriented randomly within the same leaf region (Rudall et al., 2013; Figure 1). Despite a debatable function in gas exchange vs. sporophyte dehiscence in moss species (Merced, 2015; Chater et al., 2016), stomatal complexes in land plants are canonically thought to serve as epidermal valves that open and close repeatedly and reversibly to respond to various stimuli in a changing terrestrial environment. For more thorough and detailed overviews on the cell differentiation and division events during stomatal development and the signal transduction networks that underlie stomatal movements, we recommend other nicely written reviews (Fan et al., 2004; Bergmann and Sack, 2007; Casson and Hetherington, 2010; Kim et al., 2010; Pillitteri and Torii, 2012; Hepworth et al., 2018). In this update, we will focus on the walls that surround guard cells and discuss their functions and dynamics during pore formation and stomatal movements.

## The primary wall

Growing plant cells are encased by a three-dimensional cell wall called the primary wall, wherein cellulose is embedded in a matrix containing hemicelluloses, pectins, and structural proteins (Somerville et al., 2004). The biosynthesis, modification, degradation, and reorganization of wall polymers and their interactions make the primary wall quite complex and dynamic (Voiniciuc et al., 2018). The composition of the primary wall is diverse across plant species. For instance, there are two major types of primary walls in flowering plants, based on the relative amounts and types of matrix polymers. Most eudicots such as *Arabidopsis thaliana* (Arabidopsis), and non-commelinoid monocots, possess a Type I cell wall, with xyloglucan being the predominant hemicellulose and pectins composing 20–35% dry weight of the wall; in contrast, Type II cell walls are typical in commelinoid monocots such as grasses, and contain xylans and mixed-linkage glucans as the major hemicelluloses and much less pectin than Type I cell walls (Jones et al., 2005; Vogel, 2008).

For a given plant cell, wall composition undergoes spatiotemporal changes during cell development and differentiation, with older polymers such as middle lamellar pectins being deposited earlier and thus being farther from the plasma membrane, and nascent materials being laid down later and thus being closer to the cell surface (Keegstra, 2010). Cell growth in the short term, such as over a few minutes, can involve large-scale reorientations of wall components (Anderson et al., 2010).

Cellulose is synthesized at the cell surface by plasma membrane-localized cellulose synthase complexes (CSCs) (Paredez et al., 2006). CSCs move along linear trajectories that co-align with cortical microtubules (MTs), but the presence of MTs is not a prerequisite for CSC motility (Paredez et al., 2006). Cellulose is the most ordered wall polymer and is often oriented transversely to the growth axis of a cell, providing tensile strength to the wall (Green, 1962). Hemicelluloses (e.g., xyloglucan) and pectins are synthesized in the Golgi and secreted to the apoplast (Wolf et al., 2009; Pauly and Keegstra, 2016). Xyloglucan can intertwine with cellulose, forming junctions that serve as mechanical hotspots for wall loosening (Park and Cosgrove, 2012a,b). Xyloglucan in extended conformations can also bind to the hydrophobic faces of cellulose (Zheng et al., 2018).

**Figure 2:**
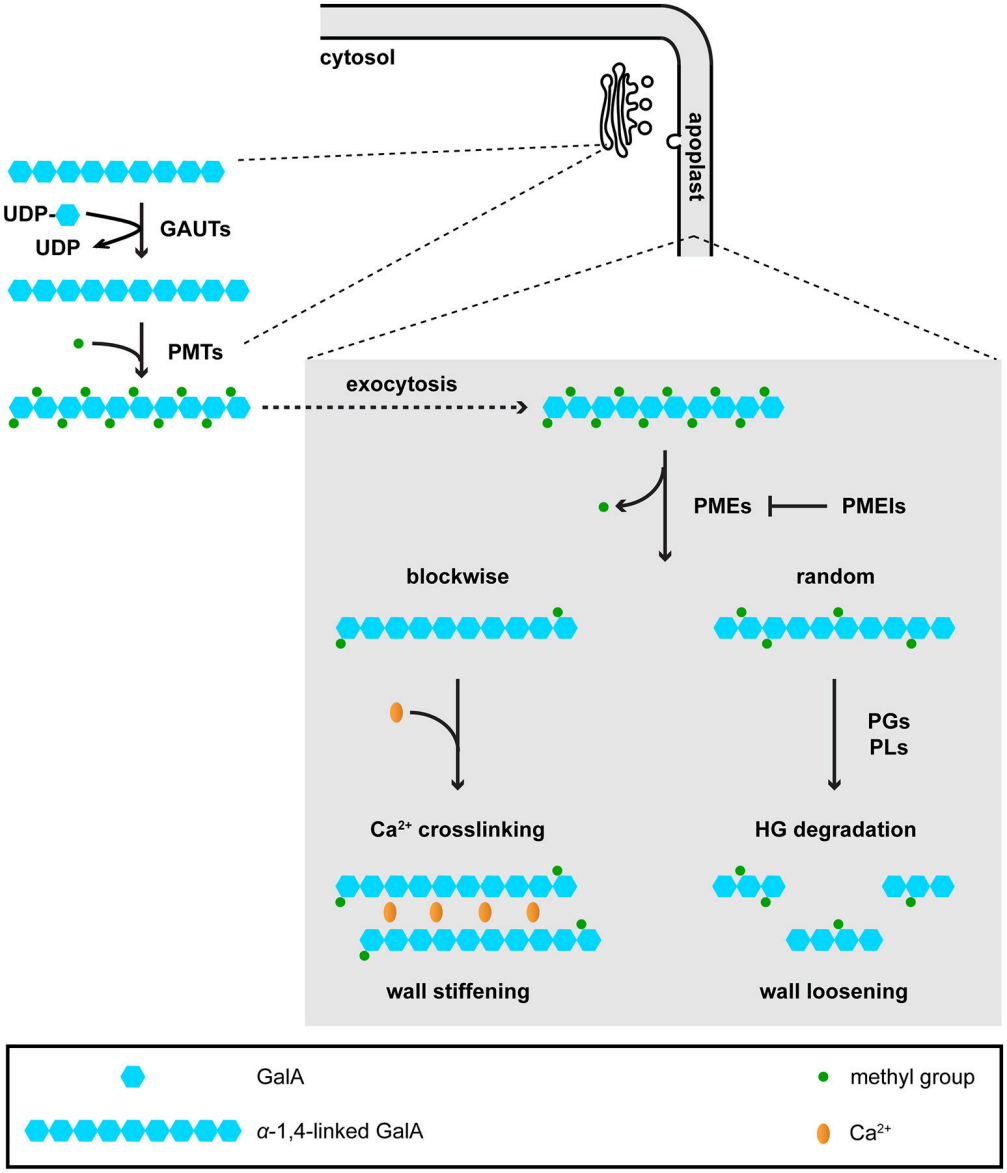
Homogalacturonan (HG) is synthesized in the Golgi, and is de-methyl-esterified and degraded in the apoplast. In the Golgi, galacturonosyltransferases (GAUTs) transfer galacturonic acid (GaiA) residues onto existing a-1,4-linked GalA chains. Pectin methyltransferases (PMTs) add methyl groups onto GalA residues. Although it is currently unknown whether PMTs function after GAUTs or PMTs and GAUTs act as a protein complex, the first scenario is shown in the figure. Highly methyl-esterified HG is then exocytosed to the apoplast, where it is de-methyl-esterified by pectin methyl-esterases (PMEs). De-methyl-esterified HG can be crosslinked by Ca^2+^, or subject to degradation by polygalacturonases (PGs) and pectate lyases (PLs).

Pectins are structurally complex polymers composed of the following domains: homogalacturonan (HG), rhamnogalacturonan-I (RG-I), rhamnogalacturonan-II (RG-II), xylogalacturonan, and apiogalacturonan (Mohnen, 2008). HG is the simplest and most abundant pectin domain. HG is synthesized and methyl-esterified in the Golgi by galacturonosyltransferases (GAUTs) and pectin methyltransferases (PMTs), respectively (Mohnen, 2008; Wolf et al., 2009). Highly methyl-esterified HG is exocytosed to the wall where it is then de-methyl-esterified by pectin methylesterases (PMEs) (Wolf et al., 2009). The methyl-esterification status of HG is also affected by endogenous pectin methylesterase inhibitors (PMEIs), which antagonize the activity of PMEs (Jolie et al., 2010). Different de-methyl-esterification patterns can lead to opposing effects on wall mechanics: blockwise de-methyl-esterification usually facilitates HG crosslinking via Ca^2+^, thus contributing to wall stiffening, whereas random de-methyl-esterification makes HG susceptible to degradation by polygalacturonases (PGs) or pectate lyases (PLs), resulting in wall loosening (Hocq et al., 2017; Figure 2). In model species such as Arabidopsis, genes encoding these pectin-modifying and -degrading enzymes all exist in large families (McCarthy et al., 2014), few of which have been functionally and/or biochemically characterized.

We have gained our knowledge of the primary wall predominantly from studies in tissue types that undergo irreversible expansion, such as roots (Anderson et al., 2010, 2012), etiolated hypocotyls (Paredez et al., 2006; Desprez et al., 2007), and shoots (Peaucelle et al., 2011; Braybrook and Peaucelle, 2013), but less so in guard cells that undergo reversible shape changes, despite the longstanding hypothesis that guard cell walls must possess unique material properties to allow for cycles of stomatal movements (Wu and Sharpe, 1979). Below, we will present an update on recent research and suggest future directions to advance our understanding of the molecular details of how guard cell walls are built to allow for fast and reversible stomatal movements.

## Composition and synthesis of the guard cell wall

The polysaccharide components of guard cell walls have been identified mostly by imaging experiments, including polarized light microscopy for cellulose, and immunolabeling coupled with fluorescence microscopy or transmission electron microscopy in thin sections for hemicelluloses and pectins (Palevitz and Hepler, 1976; Majewska-Sawka et al., 2002; Jones et al., 2003; Merced and Renzaglia, 2014; Amsbury et al., 2016; Giannoutsou et al., 2016; Shtein et al., 2017; Figure 1). These techniques, although they are not quantitative, can reveal differences in wall composition between guard cells and neighboring cells. For example, LM15 antibody-labeled xyloglucan is more enriched in guard cells than in neighboring epidermal cells in Arabidopsis (Amsbury et al., 2016), and LM6-labeled 1,5-α-L-arabinan is present in guard cells but not subsidiary cells in *Zea mays* (Giannoutsou et al., 2016). Polysaccharide components of the guard cell wall that are conserved across species include cellulose, HG, and RG-I. Pectic arabinan, in particular, has been demonstrated to maintain the flexibility of guard cell walls in various species, since exogenous treatment with arabinanase in epidermal strips prevents stomatal opening or closure in species such as *Commelina communis* and *Vicia faba* (Jones et al., 2003, 2005).

Cell wall structural proteins have also been found to be present in the guard cell wall by functional characterizations or immunolabeling approaches. In Arabidopsis, *FUSED OUTER CUTICULAR LEDGE1* (*FOCL1*) encodes a putative cell wall glycoprotein that is required for the formation of the stomatal outer cuticular ledge (Hunt et al., 2017). Plants lacking *FOCL1* have larger stomata and are impaired in controlling stomatal aperture and transpiration rate (Hunt et al., 2017), suggesting that wall structural proteins and cuticular ledges might affect stomatal dynamics. In *Z. mays*, arabinogalactan proteins have been detected in the walls encasing guard cells, but not in subsidiary cells (Giannoutsou et al., 2016).

Despite the visualization of representative wall components, a global, quantitative analysis of guard cell wall composition is still missing. This is largely due to technical difficulties in isolating and enriching enough guard cell wall materials for quantitative compositional assays. Knowing the relative amount of each wall component will aid the comparison of wall constitution between guard cells and other cell types, between dicots and monocots, and between wild type and mutant plants.

Guard cell walls are synthesized and deposited in the apoplast during stomatal development (Movie S1). As a result, their thickness gradually and differentially increases at different regions as stomata mature, with outer and inner periclinal walls eventually being thicker than ventral and dorsal walls (Zhao and Sack, 1999; Merced and Renzaglia, 2014; Figure 1). However, the molecular details of how these differentially thickened walls are synthesized and how their synthesis is spatially controlled are not clearly understood, raising several questions, e.g., which glycosyltransferases are expressed during stomatal development?; how are their activities spatiotemporally regulated?; and is there a bias of their subcellular localization at different regions within a guard cell? Transcriptomic datasets of stomatal lineage cells (Hachez et al., 2011; Adrian et al., 2015) are open resources for the search of genes encoding such glycosyltransferases, but proteomic analyses will be required to globally predict their activity levels at each stage of stomatal development.

Cellulose is actively synthesized in young guard cells, and is likely to contribute to the build-up of wall strength to withstand the high turgor pressure inside a guard cell. In Arabidopsis, although genes encoding primary wall-associated cellulose synthases (CESAs) are not highly expressed in stomatal lineage cells (Adrian et al., 2015), fluorescent protein (FP)-tagged CESAs in guard cells in young tissues are actively moving along linear trajectories, which mirror the distribution pattern of cortical MTs that radiate out from the stomatal pore (Rui and Anderson, 2016). Upon a short-term dark treatment, the co-localization between FP-CESAs and MTs is reduced in guard cells in young tissues, suggesting that some CSCs might dissociate from MT “rails” during stomatal closure (Rui and Anderson, 2016). Future studies of other glycosyltransferase families such as the cellulose synthase-like C (CSLC) family and the GAUT family, which are required for the synthesis of xyloglucan and pectins, respectively (Cocuron et al., 2007; Mohnen, 2008), will shed light on how matrix polysaccharides are produced during stomatal development.

## Organization of the guard cell wall

Some components of the guard cell wall are conserved across terrestrial plants, but their distribution patterns can be distinct in different species. For example, in ferns, dicots, and some monocots where stomata are kidney-shaped, cellulose exhibits an overall radial arrangement (Palevitz and Hepler, 1976; Fujita and Wasteneys, 2014; Rui and Anderson, 2016; Shtein et al., 2017), whereas in grasses where stomata are dumbbell-shaped, radially oriented cellulose is evident only in the polar regions (Shtein et al., 2017; Figure 1). Compared to cellulose, there are fewer studies on the spatial organization of matrix polysaccharides in guard cells, although stretches of de-methyl-esterified HG have been reported to be diffusely distributed in the periclinal wall, but enriched at the polar ends of guard cell pairs in Arabidopsis (Carter et al., 2017; Rui et al., 2017; Figure 1).

Visualization of the organization of the guard cell wall is challenging, partly due to the particularly thick cuticles that prevent penetration of many probes (Voiniciuc et al., 2018) and the projection or two-dimensional information gained by some imaging approaches such as polarized microscopy, field emission scanning electron microscopy (FESEM), and immunolabeling in thin sections. To address these issues and to learn how individual components of guard cell walls are distributed in 3D, one direction is to develop and apply a library of small fluorescent dyes (Anderson and Carroll, 2014) that can penetrate the cuticle and bind to specific wall components in guard cells. Dyes that are compatible with super-resolution imaging techniques such as structured illumination microscopy (SIM) (Gustafsson, 2005) and stochastic optical reconstruction microscopy (STORM) (Huang et al., 2008) would facilitate more finely detailed investigations of cell wall organization in intact, hydrated guard cells. Results from these dye-based imaging experiments should be interpreted with the caution that the dye might alter the function of the wall component to which it binds.

**Figure 3:**
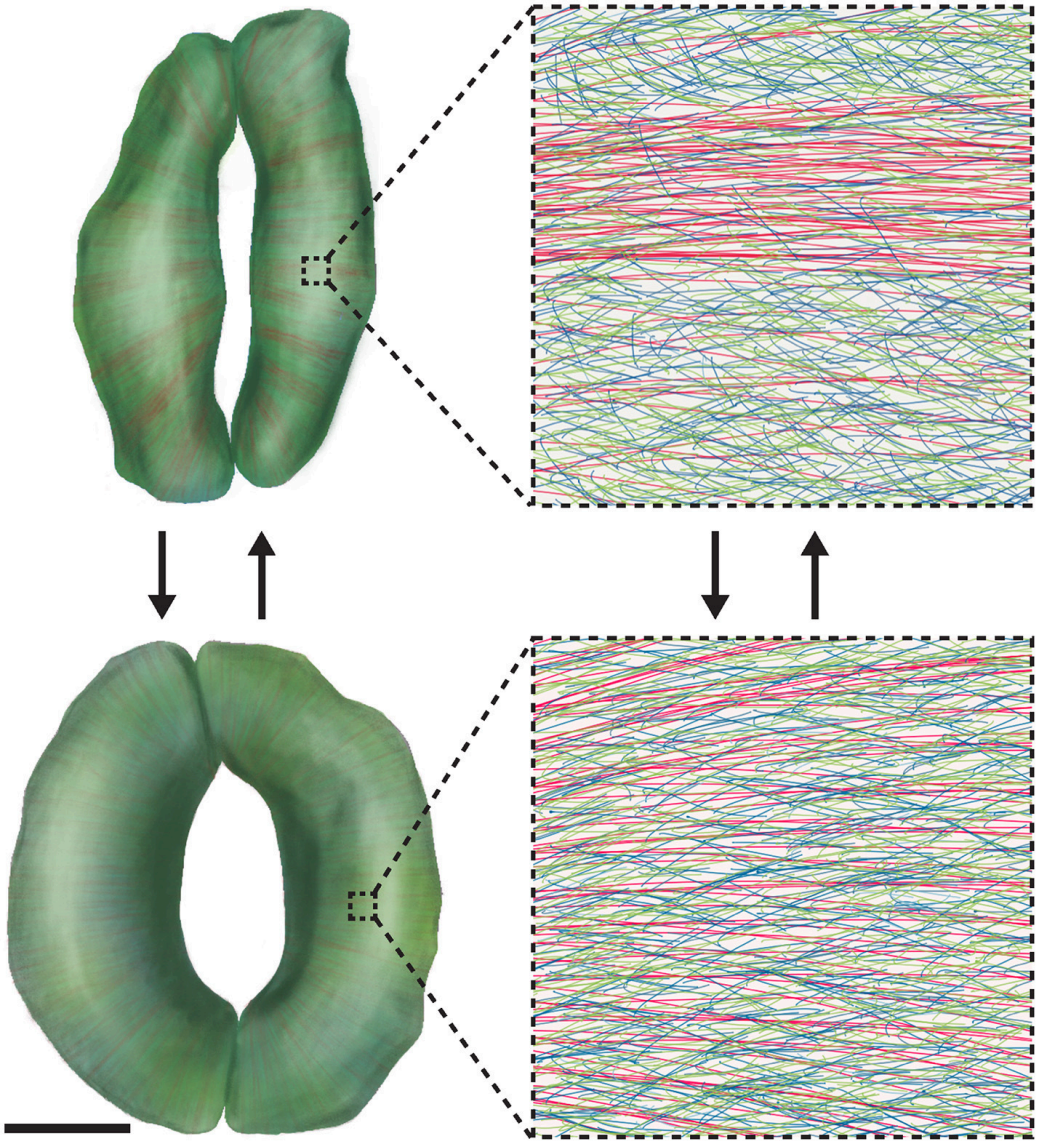
Wall Reorganization in Guard Cells Might Underlie Stomatal Dynamics. Mature guard cells might use wall materials that have been deposited to meet their functional requirement for repeated elastic deformation. 3D renderings of closed **(Top)** and open **(Bottom)** stomatal complexes are shown on the left, with cellulose shown in red, xyloglucan shown in green, and pectins shown in blue. Note that 3D renderings were made via computerized segmentation of 3D microscopic images of actual guard cells. Scale bar for images on the left is 5 μm; insets on the right show the detailed molecular organization of guard cell walls in 1 × 1 μm regions. During stomatal opening, cellulose becomes diffusely distributed, and pectic homogalacturonan networks are uncoupled. During stomatal closure, cellulose becomes bundled and pectic homogalacturonan is crosslinked by Ca^2+^. See also Movie S1.

Given that stomata open and close on a time scale of minutes and that synthesizing and/or degrading substantial amounts of wall components during every cycle of stomatal movement would be metabolically expensive (Zhang et al., 2011), one might wonder which dynamic process(es) occur in the guard cell wall to allow for rapid changes in stomatal shape. One hypothesis we have raised is that stomatal movement is accompanied by the dynamic reorganization of wall components in guard cells (Figure 3; Movie S1). One piece of data that supports this hypothesis is that cellulose microfibrils in intact guard cells of Arabidopsis exhibit a relatively even distribution when stomata are open, but become more bundled and evidently fibrillar when stomata are closed (Figure 3; Movie S1) (Rui and Anderson, 2016). Such a change in cellulose organization is aberrant in the *CELLULOSE SYNTHASE3* (*CESA3*) mutant, *cesa3*^*je*5^, that is deficient in cellulose (Desprez et al., 2007) and in a double mutant that lacks the expression of *XYLOGLUCAN XYLOSYLTRANSFERASE1* (*XXT1*) and *XXT2, xxt1 xxt2*, which is deficient in xyloglucan (Cavalier et al., 2008). In addition, stomatal apertures during stomatal movements are larger in *cesa3*^*je*5^ mutants but smaller in *xxt1 xxt2* mutants compared to wild type controls (Rui and Anderson, 2016). These observations suggest that the construction of a wall that facilitates cellulose reorganization and proper control of stomatal aperture depends on sufficient levels of cellulose and xyloglucan (Movie S1) (Rui and Anderson, 2016). In addition to cellulose reorganization, we also proposed that during stomatal movements, pectins might undergo remodeling from being un-crosslinked in the open state to crosslinked in the closed state (Figure 3; Movie S1) (Rui et al., 2017), a process that should be distinguished from the metabolic turnover of pectins (i.e., their synthesis, deposition in the apoplast, and degradation). To further test the above hypotheses, high-resolution imaging of multiple wall constituents in living guard cells during the movements of individual stomata will be needed to reveal and quantify any spatiotemporal changes in nanoscale wall organization.

## Post-synthesis modification of the guard cell wall

Comparative transcriptomic analyses between wild type Arabidopsis and mutants lacking or overexpressing a transcription factor that governs cell differentiation during stomatal development such as *STOMATAL CARPENTER1* (*SCAP1*) (Negi et al., 2013) or between different developmental stages in the stomatal lineage (Adrian et al., 2015) make possible the identification of genes encoding cell wall-modifying proteins that are likely to function in stomata. For example, *PECTIN METHYLESTERASE6* (*PME6*) is downregulated in a loss-of-function mutant of *SCAP1*, a transcription factor that is essential for maintaining the proper shape of guard cells and controlling stomatal conductance (Negi et al., 2013). A transposon insertional mutant of *PME6* exhibits a narrower range of stomatal conductance in response to changes in CO_2_ level or light intensity, as demonstrated by two independent studies (Negi et al., 2013; Amsbury et al., 2016). Although PME6 has not yet been biochemically confirmed to act as a PME, methyl-esterified pectin epitopes are more abundant in *pme6* guard cell walls than in wild type controls, suggesting a link between cell wall composition and stomatal function in guard cells (Amsbury et al., 2016). Alternatively, cell wall-modifying genes that are more highly expressed in guard cells than in guard mother cells are candidates for functional characterizations in stomatal maturation (Adrian et al., 2015).

Genes encoding wall-modifying proteins can also be identified by screening using assays to test stomatal physiology. In a thermotolerance screen using a collection of Arabidopsis *PME* mutants, a *pme34* mutant was found to be less tolerant to heat stress than wild type controls (Huang et al., 2017). *PME34* is expressed in guard cells and encodes a biochemically active PME as indicated by changes in total PME activity *in planta* in *pme34* mutants and *PME34* overexpression lines (Huang et al., 2017). These data suggest that disrupting pectin modification by PME34 might impair water evaporation through stomata during heat stress. Because de-methyl-esterified HG produced by PMEs can have contrasting mechanical effects on the wall depending on how the de-methyl-esterification occurs (Hocq et al., 2017), it is currently unknown how these PMEs alter the biomechanics of the guard cell wall. Because PMEs enzymatically remove methyl groups from the pectin backbone in the apoplast whereas putative PMTs are localized in the Golgi (Mouille et al., 2007; Kim et al., 2015; Xu et al., 2017; Figure 2), it is unlikely that a de-methylesterification/methylesterification cycle occurs on pectins in the apoplast during fast and reversible stomatal movements in mature guard cells. Instead, the de-methyl-esterification patterns of HG generated by PME activity and their mechanical effects on the guard cell wall are more likely established during guard cell morphogenesis (Movie S1). Such effects persist in the walls of mature guard cells to allow for repetitive stomatal movements.

Other cell wall-modifying proteins act non-enzymatically, and include expansins, which can cause pH-dependent wall loosening and extension (Cosgrove, 2000). *EXPANSIN1* (*EXPA1*) is expressed in Arabidopsis guard cells, and its overexpression accelerates light-induced stomatal opening by reducing the volumetric elastic modulus of the guard cell, which likely reflects the effect of EXPA1 on the mechanical properties of the cell wall (Zhang et al., 2011). Other expansin gene candidates that might function in the guard cell wall include Arabidopsis *EXPANSIN4, EXPANSIN5* (Zhang et al., 2011), and *EXPANSIN9* (Negi et al., 2013). These experimental data open up the possibility that expansin-mediated wall loosening might be another dynamic process that acts independently or synergistically with the reorganization of wall components during stomatal movement. However, it remains to be tested what process would occur during stomatal closure to revert the loosening effect of expansin, an effect we would call “wall tightening” for which there is currently little experimental evidence.

## Degradation of the guard cell wall in relation to stomatal development and movement

Plant cell walls can be degraded by exogenous or endogenous glycoside hydrolases (GHs). Exogenous treatment with arabinanase in epidermal strips prevents stomatal opening or closure in species such as *C. communis* and *V. faba* (Jones et al., 2003, 2005). However, stomatal function remains normal in Arabidopsis mutants that lack an endogenous arabinan biosynthetic gene, *ARABINAN DEFICIENT1* (*ARAD1*), and have a 25% reduction in arabinan content in leaves (Harholt et al., 2006). Given that ARAD1 is in a subgroup of glycosyltransferase family 47 (GT47) that has seven other members (Li et al., 2004; Harholt et al., 2006), it would be interesting to see whether mutants with more severe deficiencies in arabinan biosynthesis or plants that overexpress endogenous arabinanase-encoding genes have any defects in stomatal function.

The final step of stomatal development requires partial separation of the wall between sister guard cells (Bergmann and Sack, 2007), which based on analogous cell separation events in other plant tissues (Liljegren, 2012) likely involves pectin degradation in the middle lamella. However, virtually no data have been reported to support this hypothesis, although pectic strands haven been shown to be present in newly formed stomata (Carr et al., 1980). Recently, our group characterized the function of *POLYGALACTURONASE INVOLVED IN EXPANSION3* (*PGX3*) in Arabidopsis stomata. In cotyledons, GFP-tagged PGX3 is enriched at sites of stomatal pore initiation and *PGX3* expression is associated with pore dimensions, suggesting that pectin degradation by PGX3 contributes to the controlled cell separation between sister guard cells during stomatal pore formation (Rui et al., 2017). It remains to be tested whether additional mechanisms other than pectin degradation exist to separate sister guard cells at pore initiation sites, and how pectin degradation is spatially restricted to facilitate pore formation while retaining strong connections between the ends of sister guard cells at their poles (Carter et al., 2017).

Overexpression of an apple *POLYGALACTURONASE* (*PG*) leads to malfunctioning stomata, possibly due to the presence of smaller pectins and/or holes at one or both ends of stomata in transgenic leaves (Atkinson et al., 2002), but it is unclear which phenotype is the cause of defects in stomatal function in transgenic plants. In adult true leaves of Arabidopsis, *PGX3* regulates stomatal dynamics by fine-tuning the abundance of de-methyl-esterified HG and pectin molecular size, providing a molecular explanation for how pectins maintain the flexibility of guard cell walls during stomatal movement (Movie S1) (Rui et al., 2017). In addition to PGs, it would also be worthwhile to extend functional characterizations to genes encoding other wall-degrading enzymes, such as pectate lyases and glucanases.

## Approaches to studying the mechanics of guard cell walls

In addition to conventional methods such as fluorescence/electron microscopy and functional characterization of genes to investigate the guard cell wall, there are many approaches that have been applied in the cell wall field, but have not been fully exploited to investigate the biomechanical properties of the guard cell wall in particular. Atomic force microscopy (AFM) has been used to visualize the pattern and movement of cellulose microfibrils on the nanoscale in onion epidermis (Zhang et al., 2013, 2016, 2017). Unfortunately, cellulose microfibrils in the guard cell wall cannot be directly probed by AFM due to the presence of cuticles in aerial tissues. However, stiffness distribution on the cellular scale in guard cells and neighboring pavement cells can still be revealed by AFM (Sampathkumar et al., 2014; Carter et al., 2017). Recently, Carter et al. reported that guard cells are stiffer at polar regions than along their outer periphery (Figure 1) and that exogenous PG treatment weakens polar stiffness, indicating that de-methyl-esterified pectins likely contribute to the polar stiffening of guard cells, which might help to fix stomatal poles during stomatal opening (Carter et al., 2017; Woolfenden et al., 2018).

There has been growing interest in assessing responses to mechanical stress with cellular resolution in live tissues such as Arabidopsis cotyledons (Bringmann and Bergmann, 2017; Robinson et al., 2017). Bringmann and Bergmann applied stretch forces to whole cotyledons using elastic strips and observed that the distribution of polarity markers for stomatal stem cells follows the direction of tissue-wide tensile stress (Bringmann and Bergmann, 2017). This finding should be interpreted with the caveats that polarity markers are not imaged simultaneously when stretch is applied and that the amount of force applied to the cotyledon is undefined. Recently, an Automated Confocal Micro-Extensometer (ACME) that allows real-time quantification of strain response to known stresses in 3D has been developed (Robinson et al., 2017). A future avenue to understanding the stress-strain relationship of the guard cell wall is to apply ACME in leaves or epidermal peels, with the caution that mechanical stress is applied to whole tissue rather than to individual stomata. In addition, the setup of an ACME for this purpose will require expertise and perhaps some customization.

Stomatal opening and closure are driven by turgor changes in guard cells, but direct measurement of guard cell turgor is technically challenging mainly due to the small size of guard cells and their vacuoles (Franks et al., 1995). To our knowledge, pressure values of guard cells have been determined by pressure probe in only a few species (e.g., *Tradescantia virginiana* and *V. faba*) (Franks et al., 1995, 1998, 2001), all of which have much larger stomata than Arabidopsis. Another factor limiting the wide use of the pressure probe is that the number of reported successful measurements of guard cell turgor in a given species is very small. Therefore, the development of new methodologies to either directly measure or indirectly calculate guard cell turgor will be required, which could be inspired by turgor pressure measurements in other cell types. For example, a combination of micro-indentation and osmotic treatments has recently been used to estimate turgor pressure in tobacco BY-2 cells (Weber et al., 2015). In single cells, measuring changes in cell volume or dimensions under external osmotic stress could help determine mechanical parameters of the cell, such as cell stiffness as measured in the A7 stem cell line (Guo et al., 2017), and Young's modulus of the cell wall and turgor pressure as measured in fission yeast (Atilgan et al., 2015).

An emerging tool for investigating stomatal mechanics is 3D finite element modeling, although the earliest finite element models of stomata were reported in the 1970s (Cooke et al., 1976). Thus far, all published stomatal models focus on kidney-shaped guard cells during stomatal opening (Cooke et al., 1976, 2008; Rui et al., 2016; Carter et al., 2017; Marom et al., 2017; Shtein et al., 2017; Woolfenden et al., 2017), leaving the modeling of dumbbell-shaped guard cells and stomatal closure process an uncharted area. However, results from those models lead to discrepant conclusions about which guard cell features are crucial for stomatal opening. Cooke et al. took into account neighboring cells and found that stomatal opening is a consequence of the elliptical geometry of guard cells and changes in their cross-sectional shape, whereas differential thickness of the guard cell wall and radially arranged cellulose microfibrils are not essential (Cooke et al., 1976, 2008).

More recently, Woolfenden et al. constructed stomatal models based on 2D geometric parameters of guard cells and stomatal pores in *V. faba*, and argued that circumferential reinforcement by radially oriented cellulose microfibrils is required for stomatal opening (Woolfenden et al., 2017). However, this conclusion might need to be further substantiated due to the following reasons: (1) the authors modeled guard cells with idealized geometries; (2) their experimental data in Arabidopsis were not consistent with the modeling results for *V. faba*. Using the same framework of stomatal models, Carter et al. argued that differential wall thickness plays a minimal role in stomatal opening, which is consistent with what Cooke et al. found, and suggested that de-methyl-esterified HG-based stiffening at guard cell ends might function as “pins” to help to fix stomatal complex length during stomatal opening (Carter et al., 2017). Testing the effect of highly localized perturbation of HG de-methyl-esterification at polar regions will be useful to further test this hypothesis.

Good finite element models depend on high-quality inputs including geometry, boundary conditions, and material models (Bidhendi and Geitmann, 2017). The aforementioned experimental approaches to measure mechanical properties of the guard cell wall would be useful to improve the quality of finite element models. A step further is to adopt a multiscale, multiphysics modeling strategy and to incorporate interactions between wall polymers at the molecular scale and interactions between guard cells and neighboring cells at the cellular scale into the model.

## Perspective

The past few years of research have brought increased attention to the structural and functional complexity of guard cell walls. We predict that the dialog between experimental data generated from genetic, cytological, and biomechanical approaches, and mechanical modeling of stomata, will shed light on the following outstanding questions: (1) Is cell wall degradation an essential molecular mechanism underlying stomatal pore formation? (2) How is the enrichment of pectin-degrading enzymes and pectins at stomatal pore initiation sites determined and established during stomatal development? (3) What is the composition and architecture of the guard cell wall? (4) Is there any difference in wall composition between guard cells and neighboring cells? (5) Is there any difference in wall composition and/or architecture between kidney-shaped guard cells and dumbbell-shaped guard cells? (6) What are the turgor pressure values in guard cells at different developmental stages, at different opening/closed states, and in different plant species? (7) During leaf senescence, mesophyll cells undergo a reduction in chloroplast number and degradation of cortical MTs (Zeiger and Schwartz, 1982; Keech et al., 2010), whereas guard cells retain chloroplasts and MT network (Zeiger and Schwartz, 1982; Ozuna et al., 1985; Hurng et al., 1988; Willmer et al., 1988; Thomas et al., 1991). Although stomatal conductance diminishes (Willmer et al., 1988) and cuticular occlusions increase as leaves and plants age (England and Attiwill, 2005), do guard cell walls likewise “wear out” and fail, and can guard cells self-repair and continue to function?

Future studies on how the amazingly strong and elastic cell walls of stomatal guard cells are constructed and how their dynamics are spatiotemporally regulated will help elucidate structure-function relationships during reversible cell expansion in plants, knowledge of which can be translated into applications such as creating elastic biomimetic materials (Li and Wang, 2016) and generating crop species with improved gas exchange efficiency.

## Author contributions

YR and CA generated Movie S1 and wrote the manuscript with input from YC, BK, HY, JW, and VP. YC, BK, HY, and JW generated Figure 3. BK and JW developed the computer image segmentation algorithm for guard cell images that has made possible the 3D renderings shown in the illustrations in Figure 3.

### Conflict of interest statement

The authors declare that the research was conducted in the absence of any commercial or financial relationships that could be construed as a potential conflict of interest.
